# A Novel Multiband Fusion Method Considering Scattering Characteristic Fluctuation Between Sub-Bands

**DOI:** 10.3390/s25226888

**Published:** 2025-11-11

**Authors:** Peng Li, Ling Luo, Denghui Huang

**Affiliations:** 1 National Key Laboratory of Complex Aviation System Simulation, Chengdu 610036, China; lipeng27@cetc.com.cn; 2 Southwest China Institute of Electronic Technology, Chengdu 610036, China; luoling@cetc.com.cn; 3 School of Information and Communication Engineering, University of Electronic Science and Technology of China, Chengdu 611731, China

**Keywords:** frequency-dependent factor, GTD model, multiband fusion, scattering characteristics fluctuation, ultra-wideband

## Abstract

Multiband fusion (MF) technology can generate an ultra-wideband echo (UWBE) from multiple sub-band echoes (SBEs), thereby improving radar range resolution and enhancing target recognition capabilities. However, current MF methods generally do not account for the incoherence introduced by fluctuations in the scattering characteristics of scattering centers (SCs) across different frequency bands. This oversight can lead to degraded fusion performance. To address this limitation, a novel MF method that explicitly considers the fluctuation of SC characteristics between sub-bands is proposed in this paper. Firstly, a theoretical analysis of the additional incoherent phase term introduced by these fluctuations is conducted, which demonstrates its impact on fusion accuracy. Based on this analysis, scattering centers are extracted from SBEs based on the geometrical theory of diffraction (GTD) model, and then categorized into two distinct types: intrinsic scattering centers (ISCs) and unique scattering centers (USCs). Subsequently, a new incoherent phase estimation and compensation method is proposed, leveraging this categorization to effectively mitigate the inter-sub-band incoherence. The two types of SCs are then processed through either fusion or super-resolution to generate individual UWBEs, which are finally combined to form the final UWBE. The effectiveness of the proposed method is validated using both simulated electromagnetic scattering data and static measured data. Numerical results demonstrate that the proposed method achieves significantly greater fusion accuracy compared to traditional MF approaches, confirming the practical benefits of incorporating SC fluctuation modeling into the fusion process.

## 1. Introduction

Basic radar theory shows that target range resolution depends on the bandwidth of the radar signal [1]. The relative bandwidth of the transmitting signal for traditional radars is typically less than 10% of the carrier frequency, which makes it difficult to achieve high range resolution. To address this limitation, ultra-wideband (UWB) radar signals can be obtained via two methods. One approach is to construct UWB radar hardware, which results in a significant increase in system complexity and design cost. An alternative effective method of generating the UWBE is using MF technology, which is a signal processing method that fuses two or more SBEs into UWBE, which avoids the high cost of building a real UWB radar [2,3,4,5,6], and improves target recognition capability [7,8,9,10]. In addition, recent developments such as coarray tensor train methods in bistatic MIMO radar offer new perspectives on high-resolution radar processing [11,12].

With the development of MF technology, numerous methods have been proposed, which can be summarized into two categories: model-based algorithms, including traditional spectrum estimation algorithms [13,14,15,16] and sparse representation algorithms [17,18,19,20,21,22]; and data-based algorithms [23,24]. Spectrum estimation algorithms are widely used in MF because of their low calculation complexity. However, the accuracy of the spectrum estimation algorithm degrades rapidly in low signal-to-noise ratio (SNR) environments. Several modified methods have been developed to address this shortcoming. Refs. [25,26,27] presented the modified Matrix Pencil (MP) algorithm and the modified estimating signal parameter via rotational invariance techniques (ESPRIT) algorithm to enhance the accuracy of MF in low-SNR environments. Compared to the spectrum estimation algorithms, sparse representation methods are highly effective in reducing the dimensionality of the problem and enhancing resolution at low SNR, but they often suffer from computational complexity. A novel MF method based on a small multiband-measurement matrix and a nonconvex log-sum regularization (LSR) is presented in [28], which improved the reconstruction efficiency and enhanced anti-noise performance. In contrast to model-based methods, data-based methods rely directly on measurement data. Consequently, the accuracy of MF is limited by the number of samples.

The existing MF methods assume that the scattering characteristics of the target remain consistent over the entire frequency band. However, the scattering characteristics fluctuate, and they affect the accuracy of MF. Ref. [29] presented that the resolution of fused SAR (Synthetic Aperture Radar) images is influenced by the fluctuating scattering characteristics of the target. A novel MF method considering the differences of SCs in each sub-band is proposed in [19,30], which demonstrates that the estimation accuracy of MF methods is limited by the incoherence caused by differences in the SCs between sub-bands unless these differences are eliminated. Nevertheless, although the distinction between SCs in different sub-bands is acknowledged and the SCs are categorized into two distinct types, the fluctuations of the same type of SCs are not accounted for during MF processing.

The main contributions of this paper are summarized as follows:

(1) A novel multiband fusion (MF) method is proposed that explicitly considers the fluctuation of scattering characteristics of scattering centers (SCs) across sub-bands, thereby enhancing the consistency and accuracy of fusion results.
(2) An information-entropy-based phase alignment criterion is introduced to estimate and compensate for the linear phase offsets among sub-bands, enabling accurate alignment of high-resolution range profiles (HRRPs).
(3) A new fixed-phase estimation and compensation scheme derived from pole estimation is proposed to address incoherence induced by SC fluctuations.
(4) The proposed approach achieves robust and accurate ultra-wideband echo (UWBE) reconstruction by fusing intrinsic and unique scattering centers (ISCs and USCs). Both simulation and measured experiments demonstrate that the method consistently outperforms conventional MF techniques in terms of fusion precision.

The rest of this paper is organized as follows. Section 2 presents the basic theory of MF. Section 3 elucidates the proposed method. Section 4 summarizes the validation and evaluation results, and Section 5 discusses the test results and proves that the accuracy of MF is improved by the proposed method. Section 6 concludes this paper.

## 2. The Basic Theory of Traditional Multiband Fusion Technology

Based on radar theory, the frequency response of the *i*th SBE: *S_R__i_*(*f_i_*) is described by the following GTD model:

(1) $$S_{Ri}\left(f_{i}\right)=S_{i}\left(f_{i}\right)\sum_{m=1}^{M_{i}}A_{mi}{\left(j\frac{f_{i}}{f_{0}}\right)}^{\alpha_{mi}}\exp\left(-j\frac{4\pi R_{mi}f_{i}}{c}\right).$$

where *S_i_*(*f_i_*) denotes the frequency spectrum of the transmitted signals of the *i*th radar. $f_{i}=f_{0}+n_{i}\cdot \Delta f$, where $f_{0}$ represents the start frequency, $f_{i}$ is the *n*th frequency point, and Δf is the frequency interval. $A_{mi}=\left|A_{mi}\right|\cdot \exp(j\varphi_{mi})$. The definition of the common parameters in (1) can be found in Table 1. c is the speed of light, and $j={\left(-1\right)}^{1/2}.$

Then, (1) can be rewritten as:

(2) $$S_{Ri}\left(f_{i}\right)=\sum_{m=1}^{M_{i}}A_{mi}{\left(j\frac{f_{i}}{f_{0}}\right)}^{\alpha_{m}}{S^{\prime }}_{mi}\left(f_{i}\right).$$

where:

(3) $$S_{mi}^{\prime }\left(f_{i}\right)=S_{i}\left(f_{i}\right)\exp\left(-j\frac{4\pi R_{mi}f_{i}}{c}\right).$$

Transforming (3) to the time domain based on the Fourier transform theory yields:

(4) $$S_{mi}^{\prime }\left(t\right)=s_{mi}\left(t-t_{ri}\right).$$

In (4), a time delay $t_{ri}=2R_{mi}/c$ is introduced into the transmitted signal with respect to the moment of transmission. Considering that the transmitting signal is the linear frequency modulated (LFM) signal, (4) can be rewritten as:

(5) $$S_{mi}^{\prime }\left(t\right)=rect\left(\frac{t-t_{ri}}{T_{i}}\right)\exp\left[j2\pi \left(f_{ci}(t-t_{ri})+\frac{1}{2}K_{i}{\left(t-t_{ri}\right)}^{2}\right)\right].$$

where the carrier frequency and the pulse widths are denoted by $f_{ci}$ and $T_{i}$, respectively. $K_{i}$ is the frequency-modulated rate. Therefore, the frequency response of SBEs is described as:

(6) $$S_{Ri}\left(f_{i}\right)=\sum_{m=1}^{M_{i}}A_{mi}{\left(j\frac{f_{i}}{f_{c}}\right)}^{\alpha_{mi}}\cdot F\left(s_{mi}^{\prime }(t)\right).$$

In (6), *F*(⋅) is the Fourier transform. The above echo signal is processed with mixing, low-pass filtering, and matched filtering. The spectrum of the echoed signal after signal processing can be expressed as:

(7) $$M_{i}\left(f_{i}\right)=\sum_{m=1}^{M}{\tilde{A}}_{mi}{\left(j\frac{f_{i}}{f_{0}}\right)}^{\alpha_{mi}}rect\left(\frac{f_{i}}{B_{i}}\right)\exp\left[-j2\pi t_{ri}\cdot (f_{i}+f_{ci})\right].$$

In (7), ${\tilde{A}}_{mi}=\left(1/K_{i}\right)A_{mi}$, $B_{i}$ is the bandwidth of the *i*th radar. For a real radar, not only is the time delay $t_{ri}$ introduced by the target, but a system time delay will also be introduced into the radar system itself in the process of transmitting signals and receiving. At the same time, the system hardware will also introduce an additional phase term to the radar signal. The system time delay and phase are summarized as $t_{0i}$ and $\varphi_{0i}$ for each sub-band radar.

The final SBEs for each sub-band are

(8) $$\begin{array}{l} M_{i}\left(f_{i}\right)=\sum_{m=1}^{M}{\tilde{A}}_{mi}{\left(j\frac{f_{i}}{f_{0}}\right)}^{\alpha_{mi}}rect\left(\frac{f_{i}}{B_{i}}\right)\exp\left[j\phi_{mi}\right].\\ \phi_{mi}=-2\pi (\tau_{i})\cdot f_{i}-2\pi (\tau_{i})\cdot f_{ci}+\varphi_{0i}. \end{array}$$

In (8), $\tau_{i}=t_{ri}+t_{0i}$ represents the total time delay of a target, $\phi_{mi}$ is the phase term of SBE. For two different sub-bands: *i*, *j*, the phase difference is:

(9) $$\begin{array}{ll} \Delta \phi_{m} & =\phi_{mj}-\phi_{mi}\\ & =-2\pi (\tau_{j}-\tau_{i})\cdot n\Delta f\\ & \;-2\pi [(\tau_{j}-\tau_{i})\cdot f_{0}+(\tau_{j}\cdot f_{cj}-\tau_{i}\cdot f_{ci})]+\varphi_{0j}-\varphi_{0i}.\\ & =n\gamma +\beta . \end{array}$$

where:

(10) $$\begin{array}{l} \gamma =-2\pi (\tau_{j}-\tau_{i})\Delta f.\\ \beta =-2\pi \left[\left(\tau_{j}-\tau_{i})\cdot f_{0}+(f_{cj}\tau_{i}-f_{ci}\tau_{i}\right)\right]+\left(\varphi_{0j}-\phi_{0i}\right). \end{array}$$

**Figure 1:**
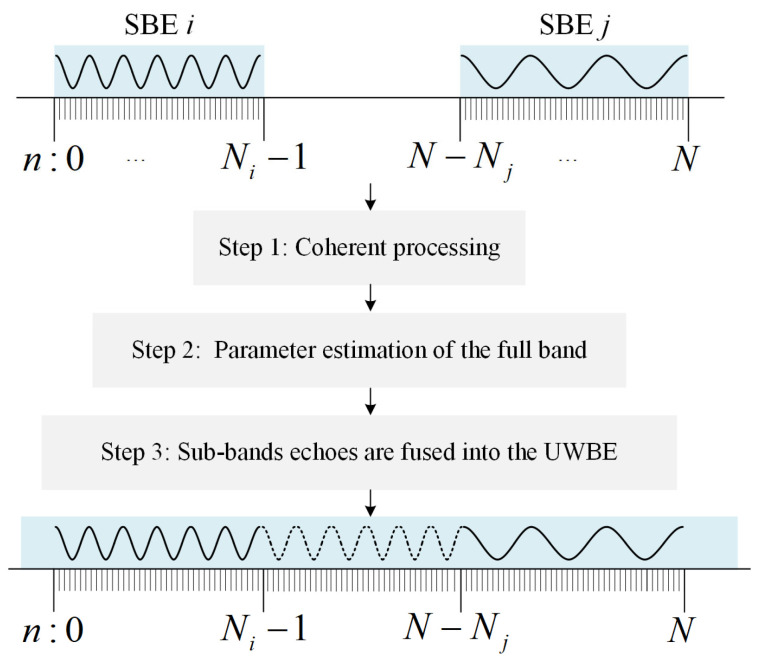
The process of MF.

As demonstrated by (9) and (10), there is a linear phase γ and a fixed phase β between two or more SBEs because of the different time delays and the phase difference between sub-bands. In [19,22,25,26,27], the traditional methods, such as the spectrum algorithms and the sparse representation algorithms, follow the three steps shown in Figure 1 to estimate and compensate the incoherent phases and generate the final UWBE. The first step is coherent processing that compensates for the linear phase and the fixed phase between SBEs. In addition, it is generally assumed that the scattering characteristics of different sub-bands are approximately in agreement, which mathematically means that the SBEs conform to a unified GTD model, and the set of parameters $\left\{A_{mi},\alpha_{mi},R_{mi}\right\}$ are kept consistent between sub-bands. Once the first step is completed, the SBEs become phase-aligned with each other. The parameter estimation of the full band is performed in the second step, and the estimated UWBE is obtained based on the parameters of the full band and the original SBEs in the third step.

## 3. The Theory and Steps of the Proposed Method

### 3.1. The Analysis of the Fluctuation of SCs

Section 2 presents the traditional MF method without considering the scattering characteristics fluctuation of SCs between sub-bands. However, refs. [4,30] demonstrated that the scattering characteristic of SC fluctuates and the sub-bands probably have different SCs, which have an impact on the accuracy of MF. Therefore, taking the different scattering characteristics of the SCs of the different sub-bands into account, the SBEs can be expressed as (11) based on (8) and (9):

(11) $$\begin{array}{ll} M_{i}(n) & =\sum_{m=1}^{M_{i}}\left|A_{mi}\right|\cdot \exp(j\varphi_{mi})\cdot {\left(j\frac{f_{0}+n\cdot \Delta f}{f_{0}}\right)}^{\alpha_{mi}}\\ & \cdot \exp\left\{-j\frac{4\pi }{c}R_{mi}\left(f_{0}+n\cdot \Delta f\right)\right\}. \end{array}$$

It takes two sub-bands, for example:

(12) $$\left\{\begin{array}{ll} M_{i}(n), & n=0,\ldots ,N_{i}-1.\\ M_{j}(n)\cdot \exp\{jn\gamma +j\beta \}, & n=N-N_{j},\ldots ,N-1. \end{array}\right.$$

In (11) and (12), $M_{i}(n)$ and $M_{j}(n)$ represent the SBE of sub-band *i* (SubI) and sub-band *j* (SubJ), respectively. The complex amplitude $A_{mi}=\left|A_{mi}\right|\cdot \exp(j\varphi_{mi})$. $N_{i}$ and $N_{j}$ represent the sampling number of SubI and SubJ, respectively. N is the sampling number of UWBE. The set of parameters $\left\{\left|A_{mi}\right|,\varphi_{mi},\alpha_{mi}\right\}$ does not remain constant at the same location $R_{mi}$ for different sub-bands due to the fluctuation in the scattering characteristics of the SCs. Meanwhile, SubI is taken as a reference, and a linear phase and a fixed phase are attached to SubJ, which must be compensated during the coherent processing. If $\Delta f/f_{0}\ll 1$, ${\left(1+n\cdot \Delta f/f_{0}\right)}^{\alpha_{mi}}$ can be rewritten as:

(13) $${\left(\frac{f_{0}+n\cdot \Delta f}{f_{0}}\right)}^{\alpha_{mi}}=\exp\left(\alpha_{mi}\ln\left(1+\frac{n\cdot \Delta f}{f_{0}}\right)\right)\approx \exp\left(\frac{\alpha_{mi}n\cdot \Delta f}{f_{0}}\right).$$

(11) is rewritten as follows:

(14) $$\begin{array}{ll} M_{i}\left(n\right) & =\sum_{m=1}^{M_{i}}\left|A_{mi}\right|\cdot \exp(j\varphi_{mi})\cdot {\left(j\right)}^{\alpha_{mi}}\exp(-j\frac{4\pi }{c}R_{mi}f_{0})\\ & \cdot \exp\left(\alpha_{mi}\frac{n\cdot \Delta f}{f_{0}}\right)\cdot \exp(-j\frac{4\pi }{c}R_{mi}n\cdot \Delta f)\\ & =\sum_{m=1}^{M_{i}}\left|A_{mi}\right|\cdot \exp(j\varphi_{mi})\cdot {\left(\exp(j\frac{\pi }{2})\right)}^{\alpha_{mi}}\exp(-j\frac{4\pi }{c}R_{mi}f_{0})\\ & \cdot {\left(\exp\left(\alpha_{mi}\frac{\Delta f}{f_{0}}\right)\cdot \exp(-j\frac{4\pi }{c}R_{mi}\Delta f)\right)}^{n}\\ & =\sum_{m=1}^{M_{i}}d_{mi}p_{mi}^{n}. \end{array}$$

In (14), $p_{mi}$ and $d_{mi}$ are the poles and the complex amplitude of the mth SC of each sub-band, respectively, which are shown in (15).

(15) $$\begin{array}{l} p_{mi}=\exp\left\{-j\frac{4\pi }{c}R_{mi}\Delta f+\alpha_{mi}\frac{\Delta f}{f_{0}}\right\}\\ d_{mi}=\left|A_{mi}\right|\cdot \exp\left\{-j\frac{4\pi }{c}R_{mi}f_{0}+j(\varphi_{mi}+\frac{\pi }{2}\alpha_{mi})\right\} \end{array}$$

Comparing the phase terms of complex amplitude $d_{mi}$ between SubI and SubJ, the scattering characteristics fluctuation of SCs superimposes additional phase terms on the traditional fixed phases β shown in (16), thus affecting the coherence between sub-bands.

(16) $$\Delta \varphi_{m}+\frac{\pi }{2}\Delta \alpha_{m}=(\varphi_{mj}-\varphi_{mi})+\frac{\pi }{2}(\alpha_{mj}-\alpha_{mi}).$$

To evaluate the effect of the scattering characteristics fluctuation of SCs on the accuracy of MF, the traditional linear phase and fixed phase are set to zero during the MF processing. The target under evaluation is assumed to have a single perfect SC with scattering characteristics fluctuations occurring across different sub-bands. Based on (14) and (15), the set of parameters $\left\{\left|A_{mi}\right|,\varphi_{mi},\alpha_{mi},R_{mi}\right\}$ for m=1, are used to represent the scattering characteristics of SubI and SubJ. Specifically, the parameters

(17) $$\left\{\begin{array}{c} Amplitude\;ratio:\left|A_{j}\right|/\left|A_{i}\right|,\\ \Delta \varphi =\varphi_{j}-\varphi_{i},\;\Delta \alpha =\alpha_{j}-\alpha_{i}, \end{array}\right.$$

are used to represent the fluctuations in scattering characteristics, where $R_{i}=R_{j}$ for the same SC. Consequently, the traditional MF method shown in Section 2 is applied to generate the UWBE. The correlation coefficient (CORC) is applied to evaluate the effect of the scattering characteristic fluctuation shown in (18).

(18) $$CORC_{\hat{M}M}=\frac{\left|{\left(\hat{M}(n)\right)}^{H}\cdot M(n)\right|}{||\hat{M}(n)|{|}_{2}\cdot ||M(n)|{|}_{2}}.$$

In (18), $\hat{M}(n)$ is the estimated UWBE. M(n) is the theoretically generated UWBE, ${\left(\cdot \right)}^{H}$ is the conjugate transpose operation, and $\|\cdot {\|}_{2}$ represents the vector 2-Norms operation. For CORC, the amplitude and phase of the reconstructed UWBE are comprehensively evaluated with the theoretically generated UWBE, and the closer the value of CORC is to 1, the closer the reconstructed UWBE is to the theoretically generated UWBE, and the higher the accuracy of the MF.

**Figure 2:**
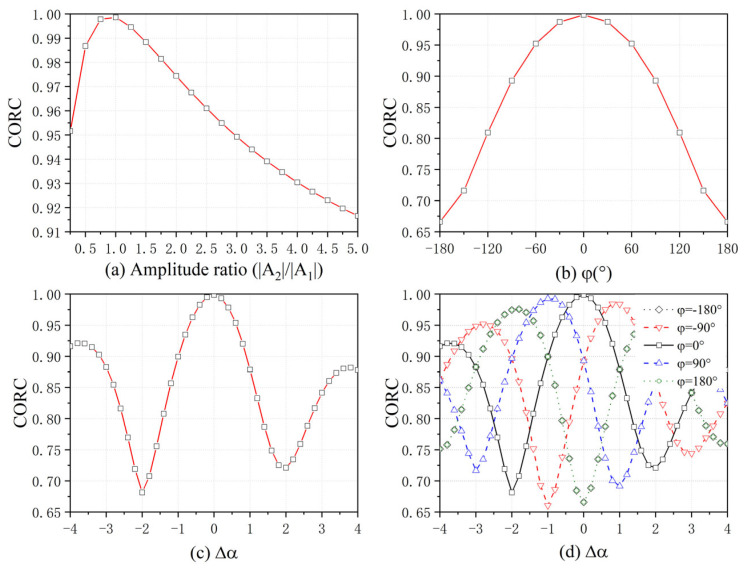
The CORC of different parameters.

Figure 2 shows the average CORC of different parameters over 100 runs of Monte Carlo tests. Figure 2a demonstrates the effect on the MF accuracy when only the absolute intensity of the scattering $\left|A\right|$ is different. When the two sub-bands have the same absolute amplitude, the CORC is infinitely close to 1, and the accuracy of MF is the highest. When only the absolute amplitude of the two sub-bands is different, it affects the absolute amplitude of the UWBE after MF, and the accuracy of MF is reduced but still maintained at a good level. This is because only the absolute amplitude is different; it does not superimpose an additional phase difference to the two sub-bands, and the two sub-bands remain phase-aligned. The effect of changing only the phase of the complex amplitude on the MF is shown in Figure 2b. The accuracy of the MF is the highest when the phase difference of the complex amplitude between two sub-bands is 0, and reaches its lowest when the phase difference varies from 0 to the phase inversion of the two sub-bands.

Meanwhile, the typical value of α with different scattering structures is [−1,−0.5,0,0.5,1]. In real scenarios, the value of α continues beyond five values. Figure 2c shows the effect of only the α difference on the accuracy of the MF. As can be seen from (16), the change in α results in a phase difference of Δα⋅π/2. Therefore, the phase difference of the two sub-band phases reaches ±π when Δα=±2, and the accuracy of the MF is at its lowest value. When Δα=0, two sub-bands are coherent with each other, and the MF accuracy is at its highest. The scattering type α also reflects the frequency dependence of the target scattering intensity, so the change of α also affects the amplitude distribution of the sub-bands, which further affects the performance of the MF.

As shown in Figure 2d, the accuracy of MF is the highest when Δα and Δφ are both 0. At the same time, when the complex amplitude phase changes, it will shift the MF accuracy curves with the change of Δα. This is because the change in the phase of the complex amplitude Δφ and the change in the scattering type Δα jointly affect the phase distribution between the sub-bands, resulting in a phase difference of Δφ+Δα⋅π/2 shown in (16). When the two terms cancel each other out, the two sub-bands will remain in phase alignment.

### 3.2. The Steps of the Proposed Method

The above analysis shows how the fluctuation of the scattering characteristics superimposes additional phase differences on the traditional fixed phases, thus affecting the MF accuracy. In this paper, we propose a new MF method that takes into account the additional phase difference due to the fluctuation of the target scattering characteristics and compensates for it using the SCs, instead of the consideration of only the traditional linear phase and the fixed phase. The accuracy of MF is thereby further improved.

As with the traditional model-based methods [13,14,15,16,17,18,19,20,21,22], the proposed method shown in Algorithm 1 is introduced with two SBEs ($M_{i}(n),M_{j}(n)$). It is known that when there is a linear phase difference between the two sub-bands, the HRRPs of the sub-bands will be shifted to the left or right. Meanwhile, according to [30], there may be differences in the SCs between the two sub-bands, so it is necessary to categorize the commonalities and differences. A method for categorizing the SCs is presented in [25], which achieves good MF results. However, if the effect of the linear phase on the HRRP is taken into account, which causes the HRRP of the two sub-bands to be far apart, the effective scattering center classification is not obtained. Therefore, it is necessary to compensate for the linear phase before performing the classification.
**Algorithm 1:** Proposed MF method**Input:**Incoherent SBEs: $M_{i}(n),\;M_{j}(n).$
**Step 1:**Estimate the linear phase $\hat{\gamma }$ between two SBEs and compensate for it to $M_{j}(n)$, obtain ${\hat{M}}_{j}(n)$.**Step 2:**Extract SCs from $M_{i}(n)$ and ${\hat{M}}_{j}(n)$ based on the GTD model, obtain the poles and the complex amplitude: $\left\{p_{mi},d_{mj}\},\{p_{mj},d_{mj}\right\}$ and categorize the SCs into two types: ISCs: $\left\{p_{miISC},d_{miISC}\},\{p_{mjISC},d_{mjISC}\right\}$ and USCs: $\{p_{miUSC},d_{miUSC}\},$
$\{p_{mjUSC},d_{mjUSC}\}.$
**Step 3:**For ISCs, calculate the new fixed phase ${\hat{\beta }}_{mISC}$ for each ISC and compensate for it to each ISC of SubJ, obtain the new complex amplitude: ${\hat{d}}_{mjISC}$.**Step 4:**Reconstruct the SBEs of ISCs based on the $\left\{p_{miISC},d_{miISC}\right\}$ and $\left\{p_{mjISC},{\hat{d}}_{mjISC}\right\}$ to obtain $M_{iISC}\left(n\right)$, $M_{jISC}\left(n\right)$.**Step 5:**Applying the second step and the third step of MF to generate the UWBE of ISCs.**Step 6:**For USCs, apply the band extrapolation to generate the corresponding UWBEs of USCs for each sub-band.**Step 7:**Add the UWBE of ISCs and UWBE s of USCs to generate the final estimated UWBE.**Output:**The final estimated UWBE.

Step 1: The estimation and compensation of the linear phase between the sub-bands. The information entropy minimization criterion is applied to estimate the linear phase, avoiding the order and phase calculation error of the traditional method shown in [10,21], etc. Assume that the real linear phase between the two sub-bands is γ and ${\hat{\gamma }}_{k}\in [-\pi ,\pi ]$ represents the compensated linear phase.

Take SubI for a reference, compensating for ${\hat{\gamma }}_{k}$ to SubJ obtain: ${\hat{M}}_{jk}\left(n\right)=M_{j}\left(n\right)\cdot \exp(-jn{\hat{\gamma }}_{k})$. Then, the HRRP of ${\hat{M}}_{jk}\left(n\right)$ is $Y_{jk}(n)$ and the HRRP of $M_{i}\left(n\right)$ is $Y_{i}(n)$. By adding the amplitudes of $Y_{jk}(n)$ and $Y_{i}(n)$, the amplitude sequence of the HRRP can be obtained as

(19) $$Y_{k}\left(n\right)=\left|Y_{i}(n)\right|+\left|Y_{jk}(n)\right|.$$

The HRRP magnitude distribution can be calculated by the following equation:

(20) $$P_{kn}={\left|Y_{k}\left(n\right)\right|}^{2}/\sum_{n=1}^{N}{\left|Y_{k}\left(n\right)\right|}^{2}.$$

The information entropy $H_{k}$ can be calculated using $P_{kn}$.

(21) $$H_{k}=-\sum_{n=1}^{N}P_{kn}\ln P_{kn}.$$

When the compensated linear phase ${\hat{\gamma }}_{k}$ is traversed with a certain accuracy between [−π,π] and ${\hat{\gamma }}_{k}$ is closer to the real linear phase γ, the superimposed HRRP has the highest degree of overlap, which corresponds to the $H_{k}$ reaches of the minimum. Therefore, the desired compensated linear phase $\hat{\gamma }$ is obtained by minimizing (23).

(22) $$\hat{\gamma }=\underset{\gamma_{k}}{\arg}\min H_{k}.$$

Compensate $\hat{\gamma }$ to $M_{j}\left(n\right)$ obtain ${\hat{M}}_{j}\left(n\right)$.

Step 2: The second step is the extraction of SCs from $M_{i}(n)$ and ${\hat{M}}_{j}(n)$ based on the GTD model and the classification of the SCs. First, the root-MUSIC algorithm is used to obtain the poles and the complex amplitude [31]: $\left\{p_{mi},d_{mi}\},\{p_{mj},d_{mj}\right\}$ from $M_{i}(n)$ and ${\hat{M}}_{j}(n)$. The relative range parameters $R_{mi},R_{mj}$ of the SCs can then be obtained:

(23) $$R_{mi,j}=-\frac{\arg\left(p_{mi,j}\right)\cdot c}{4\pi \Delta f}.$$

In (23), arg(·) denotes the operation of extracting the phase of poles. Referring to the definition from [30] and based on the relative range parameter, scattering centers from different sub-bands are classified as intrinsic scattering centers (ISCs) if their relative ranges fall within the same range resolution unit. Otherwise, SCs are identified as unique scattering centers (USCs). The range resolution unit is quantitatively defined based on the sub-band bandwidth: *c*/2*B*, where c is the speed of light and *B* is the signal bandwidth. Therefore, the poles and the complex amplitudes $\left\{p_{mi},d_{mi}\},\{p_{mj},d_{mj}\right\}$ are categorized into two types: ISCs $\left\{p_{miISC},d_{miISC}\},\{p_{mjISC},d_{mjISC}\right\}$, and USCs $\left\{p_{miUSC},d_{miUSC}\},\{p_{mjUSC},d_{mjUSC}\right\}$.

Considering the scattering characteristics fluctuation of SCs, a new fixed phase compensation method based on SC is proposed in the third step. Based on the complex amplitudes of ISCs, the traditional method of calculating the fixed phase is

(24) $$\hat{\beta }=\frac{\sum_{m=1}^{\hat{O}}\left(angle\left(d_{m2ISC}\right)-angle\left(d_{m1ISC}\right)\right)}{\hat{O}}.$$

where $\hat{O}=\min(M_{1},M_{2}).$ The calculation shown in (24) has the problem of one-to-one correspondence of complex amplitude parameters between sub-bands. Compensating the fixed phase obtained by (24) to the sub-bands is equivalent to compensating the same fixed phase for all the SCs. However, the fluctuating scattering characteristics of SCs introduces an additional phase term $\Delta \varphi_{m}+\Delta \alpha_{m}\cdot \pi /2$. Thus, the fixed phase for each ISC is different, and compensating for $\hat{\beta }$ to each SC will increase the compensation error, which ultimately weakens the accuracy of the MF.

Step 3: Because of this, it is proposed to calculate the fixed phase for each ISC respectively.

(25) $${\hat{\beta }}_{mISC}=angle\left(d_{mjISC}\right)-angle\left(d_{miISC}\right)$$

The estimated fixed phase ${\hat{\beta }}_{mISC}$ shown in (25) includes both the original fixed phase β introduced by the system characteristics and the additional fixed phase term of $\Delta \varphi_{m}+\Delta \alpha_{m}\cdot \pi /2$ introduced by the scattering characteristics fluctuation of SCs. Compensation for the modified fixed phase ${\hat{\beta }}_{mISC}$ is then applied to each ISC of the SubJ.

(26) $${\hat{d}}_{mjISC}=d_{mjSC}\cdot \exp(-j{\hat{\beta }}_{mISC}).$$

Based on steps 1 to 3, the linear phase between sub-bands is first estimated to obtain $\hat{\gamma }$, which ensure that the relative range parameters of the sub-bands are aligned. Once the sub-band echoes are linear phase-aligned, the scattering centers (SCs) can be accurately extracted and classified into intrinsic scattering centers (ISCs) and unique scattering centers (USCs). Based on SCs’ classification and considering the scattering fluctuation, a fixed-phase estimation is then performed for each ISC to compensate for the scattering fluctuation across sub-bands and achieve coherent SBEs.

Step 4: Using the poles *P_miISC_*, *P_mjISC_* and complex amplitude $d_{miISC}$, compensated complex amplitude ${\hat{d}}_{mjISC}$ to reconstruct the SBEs of ISCs for SubI and SubJ.

(27) $$\begin{array}{l} M_{iISC}\left(n\right)=\sum_{m=1}^{M_{ISC}}d_{miISC}p_{miISC}^{n},\;n=0,1,\ldots ,N_{i}-1.\\ M_{jISC}\left(n\right)=\sum_{m=1}^{M_{ISC}}{\hat{d}}_{mjISC}p_{mjISC}^{n},\;n=N-N_{j},\ldots ,N-1. \end{array}$$

In (27), $M_{ISC}$ is the number of ISCs.

Step 5: Joint parameter estimation is carried out to obtain the parameters i.e., the poles ${\hat{z}}_{mISC}$ and the complex amplitude ${\hat{a}}_{mISC}$ of the full band of the ISCs. Finally, the UWBE of the ISCs is obtained.

(28) $${\hat{M}}_{ISC}(n)=\sum_{m=1}^{M_{ISC}}{\hat{a}}_{mISC}{\hat{z}}_{mISC}^{n},\;n=0,\ldots ,N-1.$$

Step 6: According to the empirical studies in [32], a 2-octave band extrapolation offers a suitable trade-off between reconstruction accuracy and numerical stability. Therefore, for the USCs of SubI and SubJ, 2-octave band extrapolation is performed, and the remaining band data are supplemented with 0. The UWBEs of USCs of SubI and SubJ are as follows:

(29) $${\hat{M}}_{iUSC}\left(n\right)=\left\{\begin{array}{ll} \sum_{m=1}^{M_{iUSC}}d_{miUSC}p_{miUSC}^{n}, & n=0,1,\ldots ,2N_{i}-1.\\ 0, & n=2N_{i},\ldots ,N-1. \end{array}\right.$$

and

(30) $${\hat{M}}_{jUSC}\left(n\right)=\left\{\begin{array}{ll} 0, & n=0,1,\ldots ,N-2N_{j}-1.\\ \sum_{m=1}^{M_{jUSC}}d_{mjUSC}p_{mjUSC}^{n}, & n=N-2N_{j},\ldots ,N-1. \end{array}\right.$$

In (29) and (30), $\left\{M_{iUSC},M_{jUSC}p_{miUSC},p_{mjUSC},d_{miUSC},d_{mjUSC}\right\}$ are the number of USCs, the poles of the USCs, and the complex amplitude of the USCs of each sub-band, respectively.

Step 7: The UWBE of the ISCs, the UWBE of the SubI USCs, and the UWBE of the SubJ USCs are added to obtain the estimated UWBE:

(31) $$\hat{M}(n)={\hat{M}}_{ISC}(n)+{\hat{M}}_{iUSC}\left(n\right)+{\hat{M}}_{jUSC}\left(n\right).$$

The original measured sub-band data are used to minimize the estimated error. The final estimated UWBE is obtained as

(32) $$\hat{M}(n)=\left\{\begin{array}{cc} M_{i}(n), & n=0,\ldots ,N_{i}-1.\\ \hat{M}(n), & n=N_{i},\ldots ,N_{j}-1.\\ M_{j}(n), & n=N-N_{j},\ldots ,N-1. \end{array}\right.$$

## 4. Results

In this section, the performance and feasibility of the proposed method are verified through various numerical simulations. First, different radar cross section (RCS) data of different targets were generated, including the simulated RCS data of target1 obtained by FEKO simulation, and the measured RCS data of target2, the manufactured 0.5 times scaled target of target1. The different RCS data were then used to generate the SBEs. Next, the data-based method [23], the traditional model-based method [25], and the method proposed in this paper were applied to estimate the UWBE. These estimates were compared with the theoretically generated UWBE. Finally, the relative root-mean-square error (RRMSE)

(33) $$RRMSE=\frac{1}{M_{monte}{\left\|M(n)\right\|}_{2}}\sum_{i=1}^{M_{i}}\sqrt{\frac{{\left\|M(n)-\hat{M}(n)\right\|}_{2}^{2}}{N}}$$

and the CORC were calculated to verify the validity and feasibility of the proposed method. In (33), $\hat{M}(n)$ and M(n) represent the estimated UWBE and theoretically generated UWBE, respectively. $M_{monte}$ denotes the number of Monte Carlo trials.

**Figure 3:**
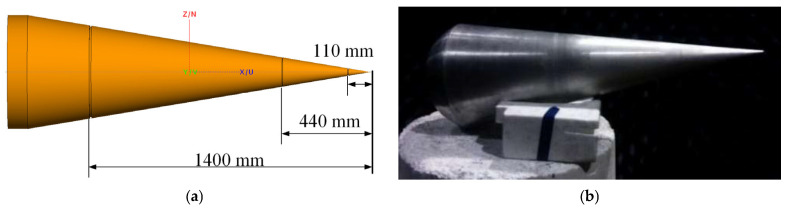
The target models. (**a**) target1: a cone with three grooves of FEKO simulation. (**b**) target2: the manufactured target of target1 at 0.5 times scale.

The simulation model of target1 is shown in Figure 3a. It is a cone with three grooves. The three-dimensional dimensions of the target1 are summarized in Table 2. The width of the first groove is 10 mm, the depth is 5 mm, and the distance from the top of the cone is 1400 mm. The second groove has a width of 4 mm, a depth of 2 mm, and is located 440 mm from the top of the cone. The third groove has a width of 6 mm, a depth of 3 mm, and is located 110 mm from the top of the cone. The FEKO 2021 electromagnetic simulation software was used to calculate the vertically polarized RCS of the target, and the observed angle was set to be 20 degrees. For target2, the vertically polarized reflected field of the target was measured in a microwave darkroom with the observed angle of incidence remaining at 20 degrees. Then, LFM signals were transmitted by two radars to obtain the echo data of the sub-band radar. The start frequencies were 4.5 GHz and 7.5 GHz for SubI and SubJ, respectively. The values of parameters used in the simulation are shown in Table 2, including the bandwidths of sub-bands, the pulse width, the linear phase, and the fixed phase between sub-bands. In addition, the theoretically generated UWBE was generated from the LFM signals with a 4 GHz bandwidth and the simulated full-band RCS data [33].

**Figure 4:**
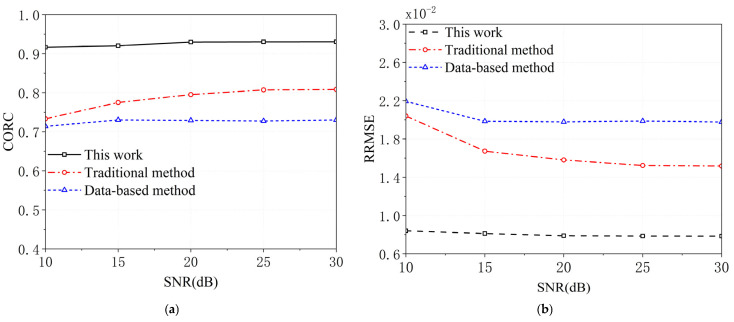
The CORC and RRMSE for target1. (**a**) The CORC, (**b**) the RRMSE.

**Figure 5:**
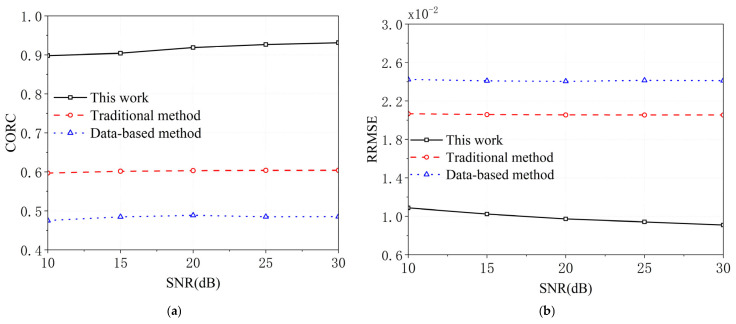
The CORC and RRMSE for target2. (**a**) The CORC, (**b**) the RRMSE.

**Figure 6:**
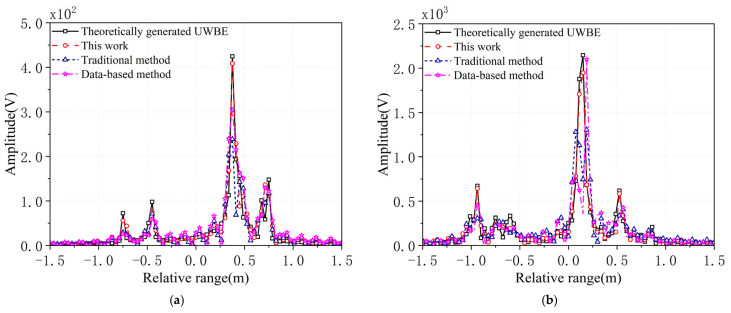
The HRRP for different methods. (**a**) The HRRP of target1, (**b**) the HRRP of target2.

**Figure 7:**
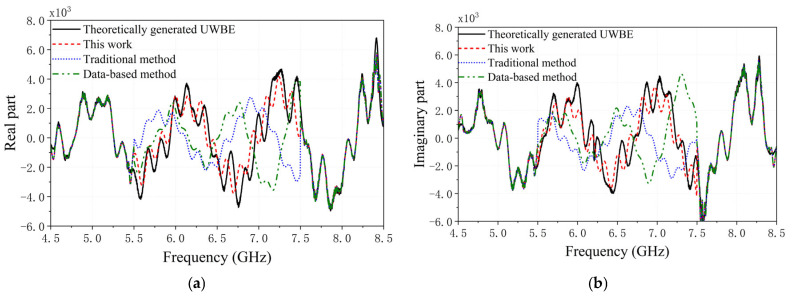
The full-band waveforms of different methods after MF for target2. (**a**) the real part, (**b**) the image part.

MF processing generates the estimated UWBE. The CORC and the RRMSE between the estimated UWBE and the theoretically generated UWBE via the proposed method, the traditional method, and the data-based method against the value of SNR under 100 runs of Monte Carlo simulations are shown in Figure 4a,b and Figure 5a,b. Figure 6a,b shows the HRRP of the proposed method, the traditional method, and the data-based method for target1 and target2. In addition, Figure 7 shows the real and imaginary parts of the UWBE obtained by different methods for target2. It can be seen that the estimated UWBE of the proposed method is also in better agreement with the theoretically generated UWBE compared to the other two methods. In addition, the UWBEs of all three methods match the theoretical values at the beginning and end of the fused band, because the original measured sub-band data at the band edges are directly utilized to minimize the reconstruction error according to (32).

## 5. Discussion

Figure 4, Figure 5, Figure 6 and Figure 7 present the results of numerical simulations. As shown in Figure 4, the proposed method achieves lower relative root mean square error (RRMSE) and higher correlation coefficient (CORC) compared to the other two methods, indicating a significant improvement in MF accuracy. Specifically, the CORC values for both targets processed using the proposed method exceed 0.9, representing an average improvement of approximately 20.9% over the other methods. Furthermore, the RRMSE achieved by the proposed method is approximately 59.5% lower, highlighting its superior fusion precision. For the model-based approaches—namely, the traditional method and the proposed method—the fusion accuracy increases with the signal-to-noise ratio (SNR). This is because a higher SNR leads to a more accurate estimation of the poles and amplitudes in the GTD model (as defined in Equation (15)), thereby improving the final MF result. In contrast, the data-driven method [23] exhibits inferior performance due to its limited number of samples, which constrains its generalization and estimation accuracy. Additionally, comparing the CORC values for Target1 and Target2, it is observed that Target2 yields slightly lower CORC values. This is primarily attributed to the fact that the measured RCS data for Target2 contains more noise, which affects the fusion performance.

As shown in Figure 6, the HRRPs generated by the traditional and data-based methods differ noticeably from the theoretical HRRP derived from the UWBE. These discrepancies include amplitude deviations, peak misalignments, and the presence of spurious peaks. In contrast, the HRRP generated using the proposed method shows strong agreement with the theoretical HRRP, demonstrating its improved fidelity in reproducing target scattering features. Moreover, Figure 7 further confirms that the UWBE estimated by the proposed method exhibits better alignment with the theoretical UWBE compared to the other two approaches, both in terms of amplitude distribution and structural consistency. In addition, the computational efficiency of the proposed method was further evaluated in comparison with the traditional multiband fusion approach. Under the same simulation and hardware conditions, the conventional method required an average runtime of 120.6 ms, whereas the proposed method took approximately 172.9 ms. The additional computational cost mainly originates from the classification of scattering centers (SCs) and the reconstruction of intrinsic scattering center (ISC) sub-band echoes, which are essential steps in achieving more accurate sub-band coherence and scattering feature preservation. Despite this increase, the overall processing time remains acceptable for practical applications, given the notable improvements in fusion accuracy and robustness demonstrated in the experimental results.

In summary, the simulation results shown in Figure 4, Figure 5, Figure 6 and Figure 7 demonstrate that the proposed method significantly enhances the accuracy of multiband fusion by explicitly accounting for the inter-sub-bands fluctuation of scattering characteristics of SCs—an aspect largely neglected in existing MF techniques. Finally, it should also be noted that the root-MUSIC algorithm is a classical spectral estimation method; its accuracy may degrade under very low SNR conditions due to inaccurate pole estimation. Therefore, the results presented in this paper are based on SNR values higher than 10 dB, which are commonly achievable in practical radar applications.

## 6. Conclusions

In this paper, a novel MF method that considers the fluctuating scattering characteristics of SCs between sub-bands is proposed to improve the estimation accuracy. The proposed method first estimates and compensates for the linear phase between sub-bands. Then, the SCs in each sub-band can be classified into ISCs and USCs based on the relative range of the SCs. Next, a new SC-based fixed phase estimation and compensation method is proposed, considering the fluctuating scattering characteristics of SCs between different sub-bands. Finally, the USCs and ISCs are either fused or super-resolved to generate corresponding UWBEs, which are added together to generate the final UWBE. Compared with the traditional MF method and the data-based method, several computer simulation results prove that the proposed method improves the accuracy of MF. Moreover, the proposed method is closer to the actual application situation and could be used in more practical MF applications.

## Figures and Tables

**Table 1:** The definitions of common parameters.

Parameters	Definition
*M_i_*	The number of SCs
*A_m_* *_i_*	The complex amplitude of the *m*th SC
$\alpha_{mi}$	The frequency dependence factor
*R_m_* *_i_*	The relative range of the *m*th SC
$\varphi_{mi}$	The phase term of the complex amplitude *A_m__i_*
γ , β	The real linear phase and fixed phase
$\hat{\gamma }$ , $\hat{\beta }$	The traditional estimated linear phase and fixed phase
${\hat{\beta }}_{mISC}$	The estimated fixed phase for ISC

**Table 2:** Parameter settings in the numerical simulations.

Parameters	Values
Fused full-band frequency:	4.5–8.5 GHz
The number of sub-bands:	2
The type of the radar signal:	LFM signal
The bandwidth of sub-bands:	1 GHz
The pulse width:	10 μs
Length of a cone in three dimensions: x, y, and z:	1820 mm, 560 mm, and 560 mm
Linear phase:	γ=π/6
Fixed phase:	β=π/2
The type of noise:	Gaussian

## Data Availability

The raw data supporting the conclusions of this article will be made available by the authors on request.

## References

[1] Bao Z., Xing M., Wang T. (2005). Radar Imaging Technology.

[2] Cuomo K.M. (1992). A Bandwidth Extrapolation Technique for Improved Range Resolution of Coherent Radar Data.

[3] Borison S.L., Bowling S.B., Cuomo K.M. (1992). Super-resolution method for wideband radar. Linc. Lab. J..

[4] Cuomo K.M., Pion J.E., Mayhan J.T. (1999). Ultrawide-band coherent processing. IEEE Trans. Antennas Propag..

[5] Piou J.E., Cuomo K.M., Mayhan J.T. (1999). A State-Space Technique for Ultra-Wide Bandwidth Coherent Processing.

[6] Cuomo K.M., Piou J.E., Mayhan J.T. (2000). Ultra-wideband sensor fusion for BMD discrimination. Proceedings of the IEEE 2000 International Radar Conference.

[7] Kang M., Ji K., Leng X., Xing X., Zou H. (2017). Synthetic Aperture Radar Target Recognition with Feature Fusion Based on a Stacked Autoencoder. Sensors.

[8] Feng S., Ji K., Zhang L., Ma X., Kuang G. (2021). SAR Target Classification Based on Integration of ASC Parts Model and Deep Learning Algorithm. IEEE J. Sel. Top. Appl. Earth Obs. Remote Sens..

[9] Zhang X., Feng S., Zhao C., Sun Z., Zhang S., Ji K. (2024). MGSFA-Net: Multiscale Global Scattering Feature Association Network for SAR Ship Target Recognition. IEEE J. Sel. Top. Appl. Earth Obs. Remote Sens..

[10] Sun Z., Leng X., Zhang X., Xiong B., Ji K., Kuang G. (2024). Ship Recognition for Complex SAR Images via Dual-Branch Transformer Fusion Network. IEEE Geosci. Remote Sens. Lett..

[11] Xie Q., Wang Z., Wen F., He J., Truong T.-K. (2025). Coarray Tensor Train Decomposition for Bistatic MIMO Radar with Uniform Planar Array. IEEE Trans. Antennas Propag..

[12] Xie Q., Wen F., Xie X., Wang Z., Pan X. (2025). Coarray Tensor Train Aided Target Localization for Bistatic MIMO Radar. IEEE Signal Process. Lett..

[13] Schmidt R. (1986). Multiple emitter location and signal parameter estimation. IEEE Trans. Antennas Propag..

[14] Yan F.G., Liu S., Wang J., Jin M. (2018). Two-Step Root-MUSIC for Direction of Arrival Estimation without EVD/SVD Computation. Int. J. Antennas Propag..

[15] Zheng M.Y., Chen K.S., Wu H., Liu X.P. (2013). Sparse Planar Array Synthesis Using Matrix Enhancement and Matrix Pencil. Int. J. Antennas Propag..

[16] Paulraj A., Roy R., Kailath T. Estimation of signal parameters via rotational invariance techniques—ESPRIT. Proceedings of the MILCOM 1986-IEEE Military Communications Conference: Communications-Computers: Teamed for the 90’s.

[17] Qu L.L., An S.M., Yang T.H., Sun Y.P. (2018). Group Sparse Basis Pursuit Denoising Reconstruction Algorithm for Polarimetric Through-the-Wall Radar Imaging. Int. J. Antennas Propag..

[18] Ning Y., Zhou F., Liu L., Bai X. ISAR Multi-Band Fusion Based on Attributed Scattering Center. Proceedings of the 2018 International Conference on Radar (RADAR).

[19] Zhu X.X., Liu L., Guo B., Hu W., Ma J., Shi L. (2021). Coherent compensation and high-resolution technology of multi-band inverse synthetic aperture radar fusion imaging. IET Radar Sonar Navig..

[20] Hu P., Xu S., Wu W., Chen Z. (2018). Sparse Subband ISAR imaging based on autoregressive model and smoothed ℓ0 Algorithm. IEEE Sens. J..

[21] Liu Q.H., He Y.X., Ding K. (2022). Complex Multisnapshot Sparse Bayesian Learning for Offgrid DOA Estimation. Int. J. Antennas Propag..

[22] Zhang H.H., Chen R.S. (2014). Coherent Processing and Super resolution Technique of Multi-Band Radar Data Based on Fast Sparse Bayesian Learning Algorithm. IEEE Trans. Antennas Propag..

[23] Liu C.L., He F., Gao X.Z. A novel coherent compensation method for multiple radar signal fusion imaging. Proceedings of the 2009 2nd Asian-Pacific Conference on Synthetic Aperture Radar.

[24] Tian J., Sun J., Wang G., Wang Y., Tan W. (2013). Multiband radar signal coherent fusion processing with IAA and apFFT. IEEE Signal Process. Lett..

[25] Zou Y.Q., Gao X.Z., Li X., Liu Y.X. (2016). A Matrix Pencil Algorithm Based Multiband Iterative Fusion Imaging Method. Sci. Rep..

[26] Zhang S., Yang J., Ge P.C. (2022). TLS-ESPRIT multiband fusion processing based on hankel matrix improvement. Electron. Opt. Control.

[27] Jiang L.B., Zheng S.Y., Yang Q.W., Zhang X.K., Wang Z. (2023). Incoherence parameter estimation and multiband fusion based on the novel structure-enhanced spatial spectrum algorithm. J. Syst. Eng. Electron..

[28] Jiang W., Huang J., Li W. (2023). A Novel Multiband Fusion Method Based on a Small Multiband-Measurement Matrix and a Nonconvex Log-Sum Regularization. IEEE Geosci. Remote Sens. Lett..

[29] Hai Y., Liu L., Li Z.Y. (2024). Optimal sub-band selection algorithm for pseudo-color image synthesis in microwave photonic SAR. J. Radars.

[30] Jiang W., Sun Y., Yan K., Li W. (2023). A Novel Multiband Fusion Method Based on Differential Processing of Scattering Centers to Eliminate Incoherence Between Sub-Bands. IEEE Geosci. Remote Sens. Lett..

[31] Zou Y., Gao X., Li X., Liu Y. A high precision GTD parameter estimation method. Proceedings of the IEEE 2015 8th International Congress on Image and Signal Processing (cisp).

[32] Suwa K., Iwamoto M. A bandwidth extrapolation technique of polarimetric radar data and a recursive method of polarimetric linear prediction coefficient estimation. Proceedings of the IEEE International Geoscience and Remote Sensing Symposium.

[33] Wang T.J., Zhang Y., Zhao H., Zhang Y.X. (2017). Multiband radar signal coherent processing algorithm for motion target. Int. J. Antennas Propag..

